# Isolation of Two Virus-Like Circular DNAs from Commercially Available Milk Samples

**DOI:** 10.1128/genomeA.00266-17

**Published:** 2017-04-27

**Authors:** Konstantina Falida, Sebastian Eilebrecht, Karin Gunst, Harald zur Hausen, Ethel-Michele de Villiers

**Affiliations:** Division of Episomal-Persistent DNA in Cancer and Chronic Diseases, Deutsches Krebsforschungszentrum, Heidelberg, Germany

## Abstract

Epidemiological data indicate a potential relationship between milk and dairy product consumption and the incidence of breast cancer, as well as neurodegenerative diseases. We report the isolation of two novel circular DNA molecules isolated from commercially available milk.

## GENOME ANNOUNCEMENT

A number of studies link the consumption of milk and dairy products with the incidence of breast cancer, as well as of neurodegenerative diseases, such as multiple sclerosis (1, 2). Eighteen novel circular DNA molecules were recently isolated from cow milk, bovine sera, and blood and tissue samples from multiple sclerosis patients (3–6). We extended these studies to the analyses of additional dairy milk samples, as well as other dairy products, i.e., six commercially available dairy milk samples and two samples each from yogurt, crème fraîche, curd cheese, and butter.

DNA was extracted with phenol-chloroform and subjected to rolling-circle amplification (RCA) using random primers. Resulting products were amplified by PCR using specific abutting primers, as previously described (3), targeting a highly conserved region in the replication gene. PCR products were cloned into pCR2.1 vector (Invitrogen) prior to sequencing by primer walking. We isolated two novel circular DNA sequences from one dairy milk sample, cow milk isolate (CMI) 5.170 (1,706 bp) and CMI5.240 (2,406 bp), sharing 89% and 68% nucleotide identity, respectively, to multiple sclerosis brain isolate (MSBI) 1.176 (1,766 bp) (3). The open reading frame (ORF) encoding a putative replication protein (324 amino acids [aa]) is highly conserved between these isolates, with only a 1-amino-acid difference between the putative Rep protein of CMI5.170, and a 3-amino-acid difference in CMI5.240 compared to that of MSBI1.176. A poly(A) for each of these ORFs is located at nucleotides (nt) 1637 to 1642 (CMI5.170) and nt 1927 to 1932 (CMI5.240). Repeat regions (22 nt × 4) are present in analogy to previous isolates (3). CMI5.240 contains an additional larger ORF (125 aa) in an antisense direction, which shares 96% amino acid identity to a similar antisense-directed ORF in CMI2.214 (3).

Furthermore, we demonstrated the presence of three previously isolated agents in additional dairy products. CMI3.168 was isolated from milk, yogurt, and curd cheese, CMI4.158 from milk, and healthy cattle blood isolate (HCBI) 6.252 from milk and yogurt (3).

### Accession number(s).

The complete sequences of CMI5.170 and CMI5.240 have been deposited in the EMBL database under the accession numbers LT715554 and LT715555, respectively.
